# Estimated summer abundance and krill consumption of fin whales throughout the Scotia Sea during the 2018/2019 summer season

**DOI:** 10.1038/s41598-024-57378-3

**Published:** 2024-03-29

**Authors:** Martin Biuw, Ulf Lindstrøm, Jennifer A. Jackson, Mick Baines, Nat Kelly, George McCallum, Georg Skaret, Bjørn A. Krafft

**Affiliations:** 1 Institute of Marine Research, Fram Centre, P.O. Box 6606, Stakkevollan, NO-9296 Tromsø, Norway; 2 NERC, High Cross, British Antarctic Survey, Cambridge, CB3 0ET UK; 3 Wildscope, Los Helechos 49, El Cuartón, Tarifa, Cádiz Spain; 4 Australian Antarctic Division, Department of Agriculture, Water and the Environment, Kingston, TAS 7050 Australia; 5 Whalephoto Marine Photography, Grünheiderstrasse 7, 17291 Oberuckersee, Germany; 6 Institute of Marine Research, Nordnes, Bergen, P.O. Box 1870, Norway

**Keywords:** Conservation biology, Population dynamics

## Abstract

Among large cetaceans in the Southern Hemisphere, fin whales were the most heavily exploited in terms of numbers taken during the period of intense industrial whaling. Recent studies suggest that, whilst some humpback whale populations in the Southern Hemisphere appears to have almost completely recovered to their estimated pre-whaling abundance, much less is known about the status of Southern Hemisphere fin whales. Circumpolar estimates in the 1990s suggest an abundance of about 5500 animals south of 60° S, while the IDCR/SOWER-2000 survey for the Scotia Sea and Antarctic Peninsula areas estimated 4670 fin whales within this region in the year 2000. More recent studies in smaller regions indicate higher densities, suggesting that previous estimates are overly conservative and/or that fin whales are undergoing a substantial increase. Here we report findings from a recent multi-vessel single-platform sightings survey carried out as part of the 2019 Area 48 Survey for Antarctic krill. While fin whales were encountered throughout the entire survey area, which covered the majority of CCAMLR Management Area 48, they were particularly abundant around the South Orkney Islands and the eastern Bransfield Strait. Large feeding aggregations were also encountered within the central Scotia Sea between South Orkney Islands and South Georgia. Distance sampling analyses suggest an average fin whale density throughout the Scotia Sea of 0.0256 ($\text{CV}=0.149$) whales per km^2^, which agrees well with recent density estimates reported from smaller sub-regions within the Scotia Sea. Design-based distance sampling analyses resulted in an estimated total fin whale abundance of 53,873 (CV = 0.15, 95% CI 40,233–72,138), while a density surface model resulted in a slightly lower estimate of 50,837 (CV: 0.136, 95% CI 38,966–66,324). These estimates are at least an order of magnitude greater than the previous estimate from the same region based on the IDCR/SOWER-2000 data, suggesting that fin whales are undergoing a substantial abundance increase in the South Atlantic. This may have important implications for the assessment of cetacean population trends, but also for CCAMLRs spatial overlap analysis process and efforts to implement a Feedback Management system for Antarctic krill. Our abundance estimate suggests an annual summer krill consumption by fin whales in the Antarctic Peninsula and Scotia Sea area of 7.97 (95% CI 4.94–11.91) million tonnes, which would represent around 20 times the total krill catch taken by the commercial fishery in Area 48 in the same season, or about 12.7% of the 2019 summer krill standing stock estimated from data collected during the same survey. This highlights the crucial importance of including cetacean krill predators in assessment and management efforts for living marine resources in the Southern Ocean, and particularly stresses the urgent need for a re-appraisal of abundance, distribution and ecological role of Southern Hemisphere fin whales.

## Introduction

During the period of industrial whaling, about 725,000 fin whales (*Balaenoptera physalus*) were harvested in the Southern Hemisphere, making it the most heavily exploited cetacean species in terms of number of animals taken^1^. As was the case with other large cetaceans, this left the stock severely depleted by the end of industrial whaling in the 1950s, and they continued to be harvested until well into the 1970’s^2^. While no systematic scientific sightings surveys were conducted during the industrial whaling period, regular summertime surveys were conducted during 1978-2004 as part of the circumpolar IDCR/SOWER program^3^. These surveys were mostly limited to south of 60° S, with poor coverage in more northerly regions where fin whales are also known to occur during summer^4,5^. Therefore, these estimates likely only represent a fraction of the total Southern Hemisphere abundance ^6^. With these limitations in mind, a reanalysis of the IDCR/SOWER survey data from 1992/93 to 2003/04 yielded an estimate for the Antarctic Ocean south of 60^∘^S of 15,109 (CV = 0.256)^7^.

**Figure 1:**
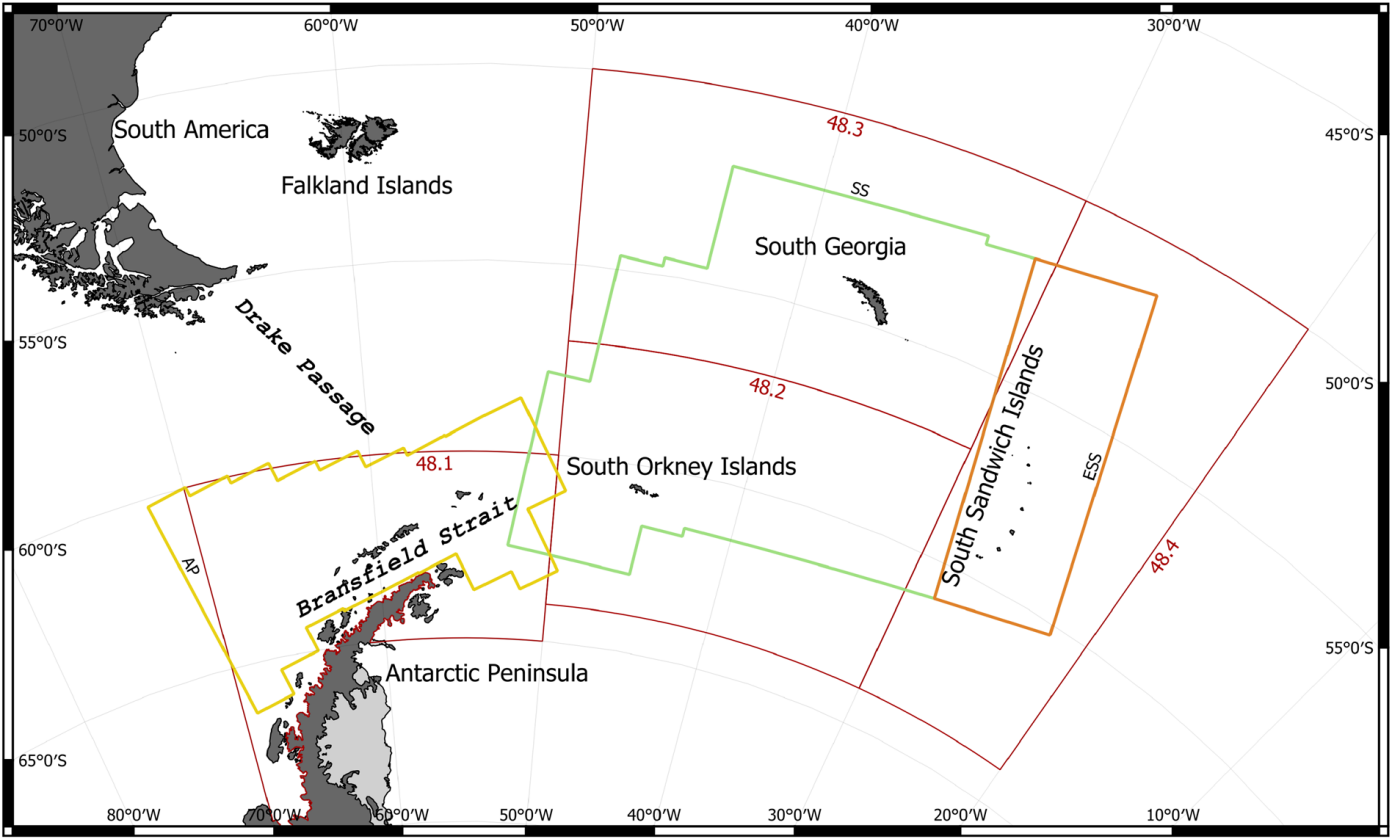
Survey area for abundance estimation of fin whales. Multicoloured thick lines correspond to strata used for estimating krill abundance from the 2019 Area 48 Survey for Antarctic krill (see Krafft et al. 2021), while thin red lines represent CCAMLR statistical subareas. 48.1–48.4. AP represents the western Antarctic Peninsula stratum, while SS and ESS represent the Scotia Sea and Eastern Scotia Sea strata respectively.

Combining abundance estimates with harvest statistics and other relevant input data, several attempts have been made to estimate population trajectories for fin whales and other large baleen whales in the Southern Ocean. For instance, Tulloch et al.^8^ recently developed a multispecies ecosystem model to estimate whale population trajectories from 1890 to present. This model estimated the pre-whaling adult female fin whale population to around 210,000 individuals in the South Atlantic sector, while the predicted 2018 female population size in the same region was 5,260 individuals. This is similar to the most recent total fin whale abundance estimate of 4,672 (95% CI 767–8577, males and females) within the Scotia Sea and Antarctic Peninsula, from a design-based analysis of the IDCR/SOWER 2000 survey data^9^ (hereafter referred to as SOWER-2000). In recent years, surveys of smaller sub-regions have yielded local abundance estimates, many of which suggest that fin whales around the Antarctic Peninsula are increasing in numbers^10,11^. Dedicated aerial surveys of the Drake Passage and Bransfield Strait (Fig. 1) carried out in 2013 suggested a minimum fin whale density (i.e. not corrected for perception and availability bias) in this region of 0.117 whales km^2^, or an estimated minimum abundance of 4,898 in an area of ~42,000 km^[212^. Based on a ship-based survey in the waters around Elephant Island and the South Orkney Islands in 2016, Viquerat and Herr^13^ estimated average minimum densities of 0.0268 ± 0.0183 and 0.0588 ± 0.0381 whales km^2^ in the two areas respectively, with minimum abundance estimates of 528 ± 362 and 796 ± 516 fin whales. These recent estimates, despite representing relatively small areas, suggest that fin whales in the SW Atlantic have increased in numbers over recent decades, something that is also supported by the increasingly common occurrence of very large fin whale feeding aggregations whales in some regions of the Southern Ocean^14,15^. However, the rate of increase remains poorly quantified ^6^, and it is not known to what extent these geographically limited estimates reflect population trends over a larger spatial scale.

It is clear that current estimates of circumpolar fin whale abundance remains highly uncertain. While efforts are currently underway to collate and analyse all available data, more survey data are urgently needed from the zone 50°–60° S to determine the potential downward bias in current estimates due to past large-scale surveys being limited to south of 60° S ^6^. The increasing demand for commercial harvesting of krill (*Euphausia superba*), especially within CCAMLR management area 48, requires careful assessment of sustainable harvest levels that also take into account the prey requirements of krill-dependent predators. Recent evidence suggests that several Southern Hemisphere populations of humpback whales (*Megaptera novaengliae*) have undergone dramatic population increases over recent decades, and some estimates suggest that they may be approaching their pre-whaling abundance in some areas^16–18^. Such population increases could have major implications for the management of krill and other living resources within the CCAMLR area. It is therefore critically important to assess if fin whales are following a similar increasing trend as several humpback whale populations, as fin whales represent a formidable krill consumer in the Southern Ocean.

Here, we report results from visual sightings obtained during the large-scale multi-vessel Area 48 Survey for Antarctic krill in the Scotia Sea, carried out in the summer of 2019 and covering most of CCAMLRs management area 48^19^. The survey was an effort to repeat the large-scale CCAMLR coordinated kill survey carried out in 2000^20^ and obtain updated biomass estimates for Antarctic krill within the same strata used during that earlier survey (Fig. 1). In this paper, we focus on describing the results from fin whale sightings, because (1) they were by far the most commonly observed large cetacean species over large parts of the survey area, allowing us to estimate abundance with reasonable uncertainty estimates, (2) fin whales may represent one of the most important (if not the most important) cetacean krill predator in the region, and (3) fin whales are some of the least well surveyed of the large baleen whales in the Southern Ocean. Given current increases in krill fishing activity and ongoing efforts by CCAMLR to carry out spatial overlap analysesfor the management of krill stocks, accounting for cetacean krill predators in the overall assessment is rapidly becoming critically important. This is particularly pertinent given recent results estimating an almost complete recovery of humpback whales to estimated pre-whaling levels in the Scotia Arc and the Antarctic Peninsula^21^.

## Materials and methods

### Field observations

Visual observations were carried out onboard three of the six vessels participating in the 2019 krill survey; the *R/V Kronprins Haakon* (KPH, observation platform height = 23.5m), the *F/V Cabo de Hornos* (CDH, observation platform height = 9.5 m) and the *RRS Discovery* (DIS, observation platform height = 25m). Surveys covered the periods 10 Jan–22 Feb (KPH), 09 Jan–11 Mar (CDH) and 03 Jan–11 Feb (DIS). Observations were taken by dedicated observers using the Normal Passing Mode (NPM) protocol^3^. Observations were carried out in sea states below Force 6, and generally covered all daylight hours (continuously modified to follow local time and daylength depending on latitude and longitude). On all vessels, only a single platform was used. On the CDH, only one dedicated observer was present onboard, while two and four dedicated observers were present on the KPH and DIS respectively. This resulted in reduced effort especially on the CDH compared to the DIS. To alleviate the problem of under-staffing, dedicated observations on CDH and KPH were generally limited to one forward quadrant (port side, or 270^∘^-360^∘^ on the KPH, starboard side, or 0^∘^-90^∘^ on the CDH, relative to the bow of the vessel), with observations in other quadrants recorded as “incidental sightings” and not included in these analyses. The highly experienced single observer on CDH worked in continuous periods for ~2 hours, with ~15-30 min breaks in-between to retain focus and concentration. On the CDH and KPH, observations were carried out from inside the bridge or an inside observation deck, and sightings were taken as voice recordings directly to disc, using the system developed for the Norwegian surveys for North Alantic minke whales^22^. This system allows the dedicated observer to record effort, weather and sightings through a handheld microphone, while maintaining full visual attention. Observations on the DIS were carried out from outside platforms by two dedicated observers (one covering each forward quadrant) and one data recorder. Data were entered into the Logger software system (http://www.marineconservationresearch.co.uk/downloads/logger-2000-rainbowclick-software-downloads/).

For each whale sighting, standard variables were recorded, including estimated radial distance, angle relative to the vessel’s heading, species, group size, swimming direction and initial cue. For these abundance estimates, only the first (primary) sighting of each animal/group was used. Once a primary sighting was recorded, the observer returned to normal scanning. However, in cases where secondary sightings of a previously sighted animal/group could be confirmed, such sightings were used to confirm species designations from the primary sighting. Using the general guidelines for IWC/SOWER cruises for species identification^3^, we recorded definite fin whale sightings as ‘fin whale’, and likely fin whale sightings as ‘like fin whale’. Distinguishing fin whales from blue and humpback whales is relatively straightforward, except at great distances in which case all sightings were commonly classed as “large baleen whales” or “large cetaceans” and thus not included in these analyses. There is greater risk of confusing fin- and sei whales, especially at greater distances. To minimise the potential bias caused by erroneously classifying a sei whale sighting as ‘like fin whale’, we paid close attention to the shape of the dorsal fin, and also the surfacing patterns in the case of repeated sightings. When confirmed repeated sightings occurred at close range, almost all sightings initially classified as ‘like fin whale’ were indeed confirmed to be fin whales. This, in combination with the relative scarcity of sei whales at these southern latitudes, misclassifications are unlikely to represent a sufficiently large number to substantially bias the fin whale density and abundance estimates by including the “like fin whale” sightings class.

Radial distance was estimated using either $7 \times 50$ reticulated binoculars or (on the KPH) 30-cm equidistant steps on a mast ladder positioned 16.6 meters forward of the observation deck. These steps corresponded to different angles of depression relative to the horizon, calibrated for the height of each observer. Essentially, this method follows the exact same logic as that of reticulated binoculars or a distance stick. Angle relative to the bow was determined using a standard angle board. Weather and sea-state were recorded every 15–30 min, and in some cases (KPH) detailed weather station data were available from the ship’s automatic data recording system.

### Data preprocessing

Data from all three vessels were combined and checked for consistency. For CDH and KPH, only data from one forward quadrant were used, ignoring incidental sightings in the alternate quadrant. The port and starboard quadrant was the primary quadrant on KPH and CDH respectively. Only sightings classed as “fin whale” (i.e. certain species ID) and those classified as “like fin whale” were used. Radial distances were converted to perpendicular distances using standard trigonometry:

$$\begin{aligned} d_{perp}=d_{rad}*sin(rad(\alpha )) \end{aligned}$$

where $\alpha$ is the angle (in degrees) of the sighting relative to the bow of the vessel. We excluded a very small number of observations with $d_{perp}>7000$ meters, as species identification for these sightings should be regarded as uncertain and leads to poor detection function model performance.

All sightings conducted while in transit to and from the three strata (e.g. across the Drake Passage or between the Falkland Islands and the South Georgia corner of the Scotia Sea stratum) were excluded from the analyses. All observations collected during dedicated effort periods within strata were used, also those carried out during connecting perpendicular legs to longer transects. Preliminary analyses confirmed that these connecting legs did not significantly change the abundance estimates, and so we opted for retaining all sightings within strata to maximize the statistical power.

### Statistical analyses

Design-based density and abundance estimates were obtained via standard line transect distance sampling, using the *Distance*^23^ and *mrds*^24^ packages for the R language and environment for statistical computing^25^. We tested both half-normal and hazard-rate detection functions, and fitted a series of models with various combinations of three candidate covariates: observer ($obs$), group size ($size$) and sea state ($wind\_speed$). For CDH and DIS, instantaneous data on sea state or wind speed were not available. For these observations, we used the closest value prior or subsequent to each such sighting. Distinct transects were defined as contiguous periods of observer(s) on effort. Effort was always discontinued whenever vessels were stationary or moving slowly during active krill trawling, and a new transect was initiated once the vessel was back on its original heading and approaching normal transect speed (~10 kn). Furthermore, transects were split whenever a vessel carried out a significant course change, using a threshold of 35^∘^. To determine the most appropriate detection function and which covariates to include, model selection was carried out based on the Akaike Information Criterion (AIC^26^). To determine the most parsimonious detection model, data from all three vessels within each of three strata used for abundance estimation (AP=Antarctic peninsula, SS=Scotia Sea, ESS=Eastern Scotia Sea, see Fig. 1) were combined. Fin whale density and abundance was then estimated separately for the three strata, as well as for all three strata combined.

In order to check for potential effects of spatial heterogeneity in our abundance estimates, we also fitted a series of density surface models (DSMs^27^), as implemented in the *dsm* package for R^28^. For simplicity, we used the detection function from the null model version of the best supported of the candidate models (i.e. without covariates for detection probability, and using the key function of the overall best supported model) of the design-based analyses. We fitted four different generalized additive models (GAMs), all including bivariate spatial smooths for coordinates. Here, latitudes and longitudes were first transformed to a Lambert Azimuthal Equal Area projection, centered around longitude 59^∘^S/46^∘^W. The simplest of these contained no environmental covariates, while the second and third also contained smooths for water depth and slope respectively, and the fourth model contained smooths for both depth and slope in addition to the bivariate smooth for coortinates. Depths were extracted from the ETOPO1 global bathymetry dataset (https://www.ncei.noaa.gov/products/etopo-global-relief-model), and slope was calculated using the *terrain* function in the *raster* package for R^29^. We split each transect into roughly 20km segments, and used a Tweedie link function to allow for overdispersion in sightings between segments. To account for potentially complex spatial patterns, we set the basis dimension to 50. We only fitted the DSMs to the entire survey area, not to each of the three individual strata.

### Krill consumption estimation

To estimate krill consumption by fin whales in the Scotia Sea region based on our abundance estimates, we adopted an approach described in^30,31^. We first simulated a set of 1000 population sizes, using our estimated abundance mean and cv from the best supported model. We used log-transformed values for the simulations to avoid non-positive simulated population sizes, and back-transformed these to real scale abundance estimates. For each simulated population size, we also simulated body masses for each individual, using random draws from a normal distribution, assuming a mean fin whale body mass of 60 tonnes and a cv of 0.2^30^.

We then used two separate approaches to estimate daily per capita rates of krill consumption. Firstly, following^30,31^, we used a generalised form of the Kleiber equation that scales average daily consumption to body mass:

$$\begin{aligned} C=\alpha M^\beta \end{aligned}$$

where $M$ is body mass (in kg) and $\alpha$ and $\beta$ are allometric scaling parameters specific to species or taxonomic groups. We used the same four sets of previously published parameter values that were used by Skern-Mauritzen et al. ^31^. These parameter values are included along with the associated consumption estimates in Table 7 in the results section.

Secondly, we used an approach based on estimated annual energy requirements to cover basal metabolic needs as well as cost of transport at some assumed average swim speed. We did not include additional metabolic costs associated with e.g. pregnancy. Basal metabolic rate (BMR)was calculated as:

$$\begin{aligned} BMR=292.9M^{0.75}\times 86.4 \end{aligned}$$

where $M$ is again body mass (in kg), and 86.4 is a multiplier converting from kJ day^-1^ to its SI unit version J s^-1^. Following^32^, we estimated the energy required for moving through water as:

$$\begin{aligned} E_{COT}=\Biggl (\frac{\lambda }{2\epsilon _A\epsilon _P}\Biggr )\rho SC_dV^3 \end{aligned}$$

where $E_{COT}$ is energy cost of transport, $\lambda$ is a ratio of active to passive drag (here set to 0.7), $\epsilon _A$ is the aerobic efficiency of turning metabolized energy into mechanical work, $\epsilon _P$ is the propulsive efficiency of converting muscle work to forward propulsion, $\rho$ is the density of seawater (assumed to be 1027 g l^-1^), $S$ is wetted surface area (in m^2^), calculated assuming an average body length of 20m and maximum circumference of $2.7\pi$, where 2.7 is a typical width measurement from overhead photogrammetric analyses of fin whales based on drone measurements carried out around the South Orkney Islands in Jan-Feb 2023 (Biuw et al. unpublished), and assuming a shape of a prolate spheroid, $C_d$ is drag coefficient (using an average value for cetaceans of 0.003, following ^33^, and $V$ is swimming speed (in m s^-1^). In order to estimate the prey consumption required to meet these energy requirements, we assumed an energy assimilation efficiency from prey of 0.9, and that 80% of the annual energy requirement is met by foraging within the high southern latitude summer feeding grounds. While there is great uncertainty regarding the summer residence time of fin whales on these feeding grounds, we have assumed a period of 120 days. Finally, to estimate the krill biomass required to meet these energy requirements, we have assumed a krill energy content of 4400 J g^-1^^34^. We present consumption estimates for each of the four sets of allometric parameters as well as that derived from annual energy requirements, providing both daily and annual (seasonal) estimates.

## Results

### Survey coverage

**Figure 2:**
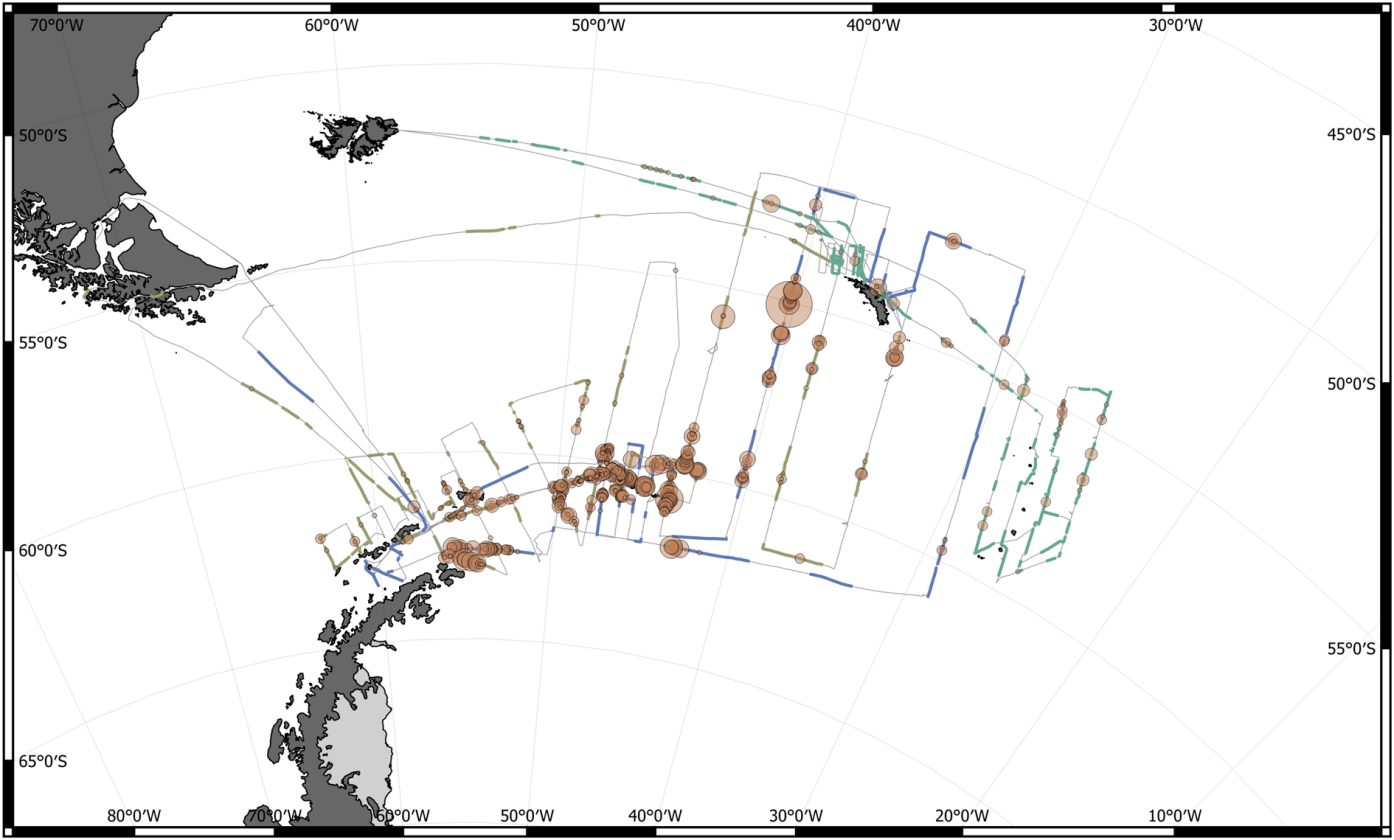
Survey lines of three vessels, R/S Kronprins Haakon (KPH), F/V Cabo de Hornos (CDH) and RRS Discovery (DIS), participating in the 2019 Area 48 survey for Antarctic krill. Bold segments represent on-effort periods for the three vessels respectively (KPH in blue, CDH in lightbrown and DIS in green). Fin whale sightings are represented by orange filled circles, with diameter proportional to relative group size.

Survey tracks, effort coverage and fin whale sightings are shown in Fig. 2. The three vessels achieved broad coverage throughout the main parts of the Scotia Sea, from the Drake Passage in the west to the South Sandwich Islands in the east. By design, the 2019 Area 48 Survey for Antarctic krill did not cover the northwestern sector of the Scotia Sea, as Antarctic krill are generally not encountered in this region north of the Polar Front.

### Sightings

When including all sightings on both sides of the vessels, 584 sightings were obtained, with an estimated 1,081 individual whales. Of these, 858 were classified as definite fin whales, while 223 were classified as likely fin whales. This constitutes by far the greatest proportion of all marine mammal sightings obtained during the survey. Overall, 36 different species categories of marine mammals were observed during the survey. there were a total of 12 definite blue whale sighting events, 6 definite sei whale sighting events, 393 definite humpback whale sighting events, and 306 unidentified large baleen whale sighting events.

While fin whales were encountered in all survey strata, the greatest concentrations were observed in the waters around the South Orkney Islands and in the southeastern part of the Bransfield Strait, off the NE tip of the Antarctic Peninsula. Regular sightings of fin whales were also obtained in the central Scotia Sea, especially on transects between the South Orkney Islands and South Georgia.

Table 1 presents an overview of the realised survey effort and number of fin whale sightings that were actually considered in the distance sampling analysis, following the previosly described subsetting procedures (see Materials & Methods).

**Table 1:** Realised effort (nm), number of sightings (groups) and number of individuals by vessel and stratum.

	Antarctic Peninsula		Scotia Sea		Eastern Scotia Sea	
	Effort (nm)	Groups (Individuals)	Effort (nm)	Groups (Individuals)	Effort (nm)	Groups (Individuals)
CDH	873	68 (108)	1305	180 (331)		
DIS			843	17 (27)	1009	25 (38)
KPH	548	28 (63)	1545	116 (198)		

While most sightings were registered as single individuals (61.4% single-animal sightings, overall mean group size: 1.78), multiple sigtings often occurred in close proximity within larger groupings, often representing feeding aggregations. Such groups were frequently observed around the South Orkney Islands, but also to the SW of South Georgia where very large feeding aggregations of fin- and humpback whales were encountered.

### Candidate detection functions

The candidate detection functions fitted to the sightings data are shown in Table 2. All models had Cramér von Mises p-values well above 0.05, suggesting they all provided adequate fits to the data^35^. Overall, based on delta AIC values, there was stronger support for hazard-rate models using some combination of the covariates sea state, observer id and group size, compared to other models. The model with greatest support was a Hazard-rate model with covariate structure ~obs + wind_speed, while the second best supported model had covariate structure ~obs. The wind speed parameter was negative (-0.0125, se=0.0094), indicating that half strip width decreased as wind speed increased. The two top models yielded very similar fin whale detection probability estimates (0.358, se=0.0128, and 0.354, se=0.0131 for the top two models respectively). By comparison, the null models (i.e. those with no covariates) had very poor support (Table 2).

**Table 2:** Model selection table of both half-normal and hazard-rate detection function models.

Key function	Formula	C-vM *p*-value	$\hat{P_a}$	se($\hat{P_a}$)	$\Delta$AIC
Hazard-rate	~obs + wind_speed	0.530	0.358	0.0128	00
Hazard-rate	~obs	0.651	0.354	0.0131	0.190
Hazard-rate	~obs + size	0.631	0.354	0.0130	1.070
Hazard-rate	~obs + size + wind_speed	0.631	0.354	0.0130	1.90
Half-normal	~obs	0.255	0.301	0.0122	4.650
Half-normal	~obs + size	0.240	0.301	0.0124	5.370
Half-normal	~obs + wind_speed	0.241	0.301	0.0122	5.650
Half-normal	~obs + size + wind_speed	0.229	0.30	0.0123	6.730
Half-normal	~vessel	0.470	0.311	0.0118	19.16
Hazard-rate	~vessel	0.178	0.373	0.0132	22.90
Hazard-rate with cosine adjustment term of order 2	~1	0.856	0.334	0.0196	26.27
Half-normal with cosine adjustment terms of order 2,3	~1	0.806	0.337	0.0210	26.46
Hazard-rate	~size	0.323	0.367	0.0134	28.32
Hazard-rate	~wind_speed	0.391	0.364	0.0135	28.43
Hazard-rate	~size + wind_speed	0.382	0.364	0.0135	29.73
Half-normal	~size	0.678	0.320	0.0097	34.39
Half-normal	~size + wind_speed	0.676	0.320	0.0099	36.39
Half-normal	~wind_speed	0.620	0.321	0.0097	36.49

C-vM *p*-value = Cramér von Mises *p*-value, $P_a$ = average detectability, se = standard error.

**Figure 3:**
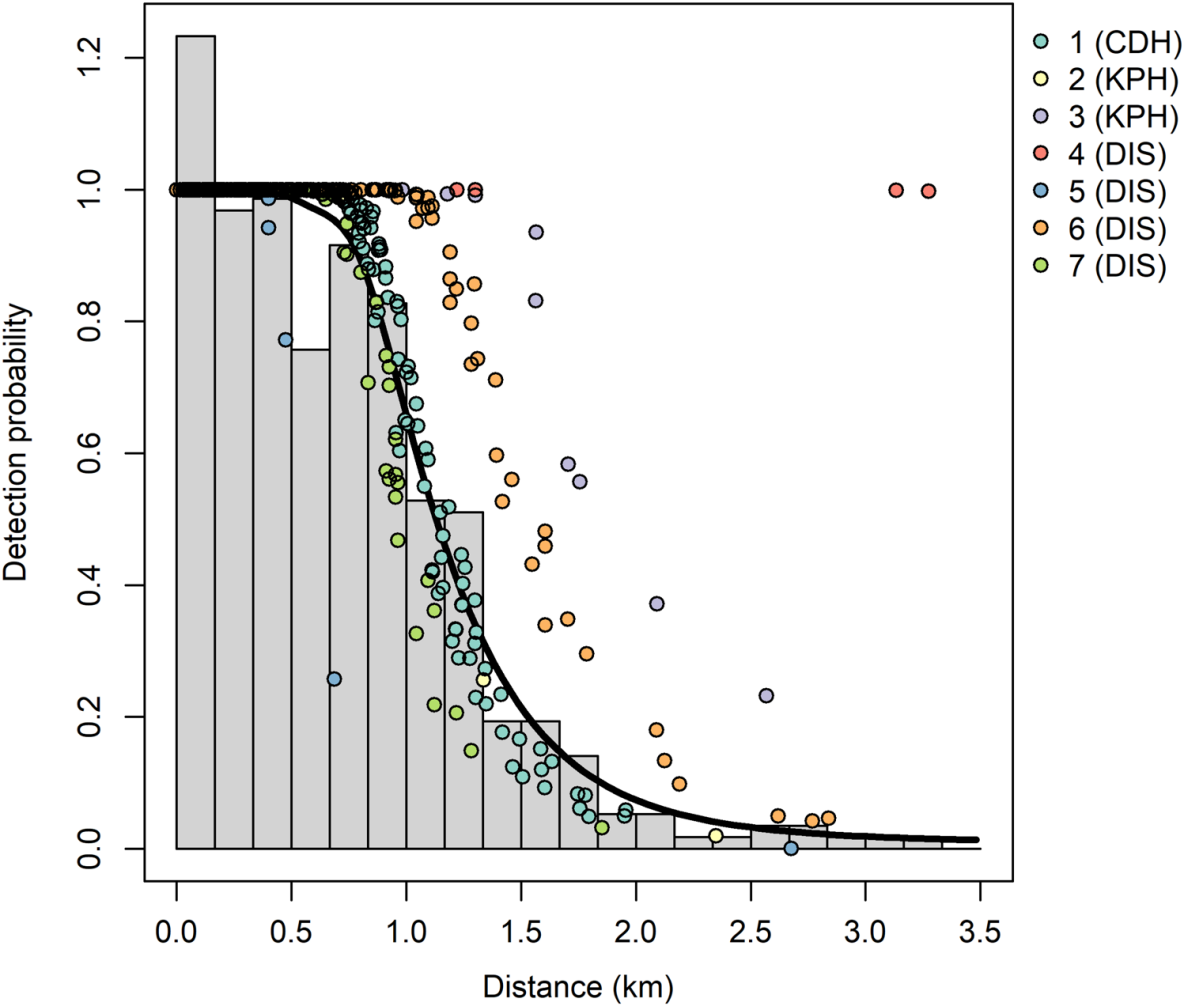
Detection function for best-fitting hazard-rate model, including effects of observer and wind speed. Colours represent individual observers.

The top model had a shape coefficient of 1.555 (se: 0.105), and scale coefficients varying substantially between observers (mean: 0.129, range: $-0.608$ to 1.567). Figure 3 clearly demonstrates this observer variation. Estimated effective half-strip width varied between observers from 0.587 to 3.5 km (mean: 1.343 km).

Table 3 presents a general model summary of the top model applied to data from each of the survey strata (AP, SS, ESS and the Complete stratum) to obtain density and abundance estimates.

**Table 3:** Number of fin whale groups ($n_s$), individuals ($n$), mean group size ($E_s$) and encounter rates per km ($ER_s$ and $ER$ for groups and individuals respectively) by strata. The number of transects is denoted by $k$. $\hat{E_s}$ represents estimated mean group size, and $se(\hat{E_s})$ its standard deviation. Total stratum area is given in km^2^ x 1000, covered area in km^2^, and effort in km.

	Area	Covered area	Effort	$k$	$n_s$	$n$	$ER_s$	$cv(ER_s)$	$ER$	$cv(ER)$	${\hat{E}}_s$	$se({\hat{E}}_s)$
AP	527	18,429	2,633	60	96	171	0.036	0.251	0.065	0.301	1.78	0.133
SS	1231	47,876	6839	170	311	552	0.045	0.167	0.081	0.160	1.77	0.077
ESS	368	13,086	1869	47	24	37	0.013	0.364	0.020	0.304	1.54	0.147
All	2101	78,586	11,227	272	413	730	0.037	0.140	0.065	0.141	1.77	0.066

Encounter rates varied between the three strata, from 0.0128 groups per km in the ESS stratum to 0.045 groups per km in the SS stratum, representing 0.0198 to 0.081 individuals per km, with estimated mean group sizes ranging from 1.54 to 1.78.

Table 4 shows the substantial differences in estimated encounter rates (${\hat{ER}}$) between the SOWER-2000 survey and the 2019 survey. In terms of the effective half-strip widths (${\hat{ESW}}$) these are variable between strata, but not consistently different between the 2000 and 2019 surveys, lending further support to fin whale densities being higher in 2019 than in 2000.

**Table 4:** Comparison of estimated encounter rates (${\hat{ER}}$) and effective half-strip widths (${\hat{ESW}}$) during the SOWER-2000 survey and the 2019 survey. To enable comparison with the SOWER-2000 data (which were presented in nautical miles), all ${\hat{ESW}}$ values from the 2019 survey were converted to nautical miles.

Survey	Stratum	${\hat{ER}}$	$cv({\hat{ER}})$	${\hat{ESW}}$	$cv({\hat{ESW}})$
2000	AP	0.015	0.540	2.26	0.096
2000	SS	0.009	0.490	0.91	0.240
2019	AP	0.120	0.557	0.69	0.096
2019	SS	0.149	0.297	0.70	0.152
2019	ESS	0.037	0.563	1.07	0.596
2019	All	0.120	0.261	0.73	0.234

### Density and abundance estimates

#### Design-based estimates

Density and abundance estimates for all strata are presented in Table 5. Fin whale densities estimated by the most supported model were 0.0323, 0.0256, 0.0065 and 0.0256 fin whales per km^2^ (CV: 0.1688, 0.3032, 0.3551 and 0.1492) in the SS, AP, ESS and Combined strata respectively. This corresponds to estimated abundances of 39,791, 13,486, 2,396 and 53,873 fin whales (95% CI 28,587–55,386, 7454–24,400, 1201–4781 and 40,233–72,138) in the SS, AP, ESS and Combined strata respectively.

**Table 5:** Estimated density (${\hat{D}}$) and abudance (${\hat{N}}$) along with their cv’s, obtained from the null and covariate models, by strata and combined. Subscript $s$ denotes groups, while no subscript denotes individuals. Included are also 95% confidence intervals of abundance estimates.

	Formula	${\hat{D}}_s$	$cv({\hat{D}}_s)$	${\hat{D}}$	$cv({\hat{D}})$	${\hat{N}}$	$cv({\hat{N}})$	$CI_{95}({\hat{N}})$
SS	null	0.019	0.177	0.035	0.171	42,507	0.171	30,427–59,384
SS	obs_wind	0.018	0.177	0.032	0.169	39,791	0.169	28,587–55,386
AP	null	0.016	0.258	0.028	0.307	14,648	0.307	8048–26,661
AP	obs_wind	0.014	0.256	0.026	0.303	13,486	0.303	7454–24,400
ESS	null	0.005	0.369	0.008	0.310	3115	0.310	1696–5721
ESS	obs_wind	0.004	0.413	0.007	0.355	2396	0.355	1201–4781
All	null	0.016	0.151	0.028	0.152	58,413	0.152	43,354–78,702
All	obs_wind	0.015	0.150	0.026	0.149	53,873	0.149	40,233–72,138

#### Density surface model estimate

Summary of DSM model results are presented in Table 6. While all four models performed relatively similarly, the model that explained the greatest proportion of the total deviance was the model including smooths for both depth and slope in addition to the bivariate smooth for location.

**Figure 4:**
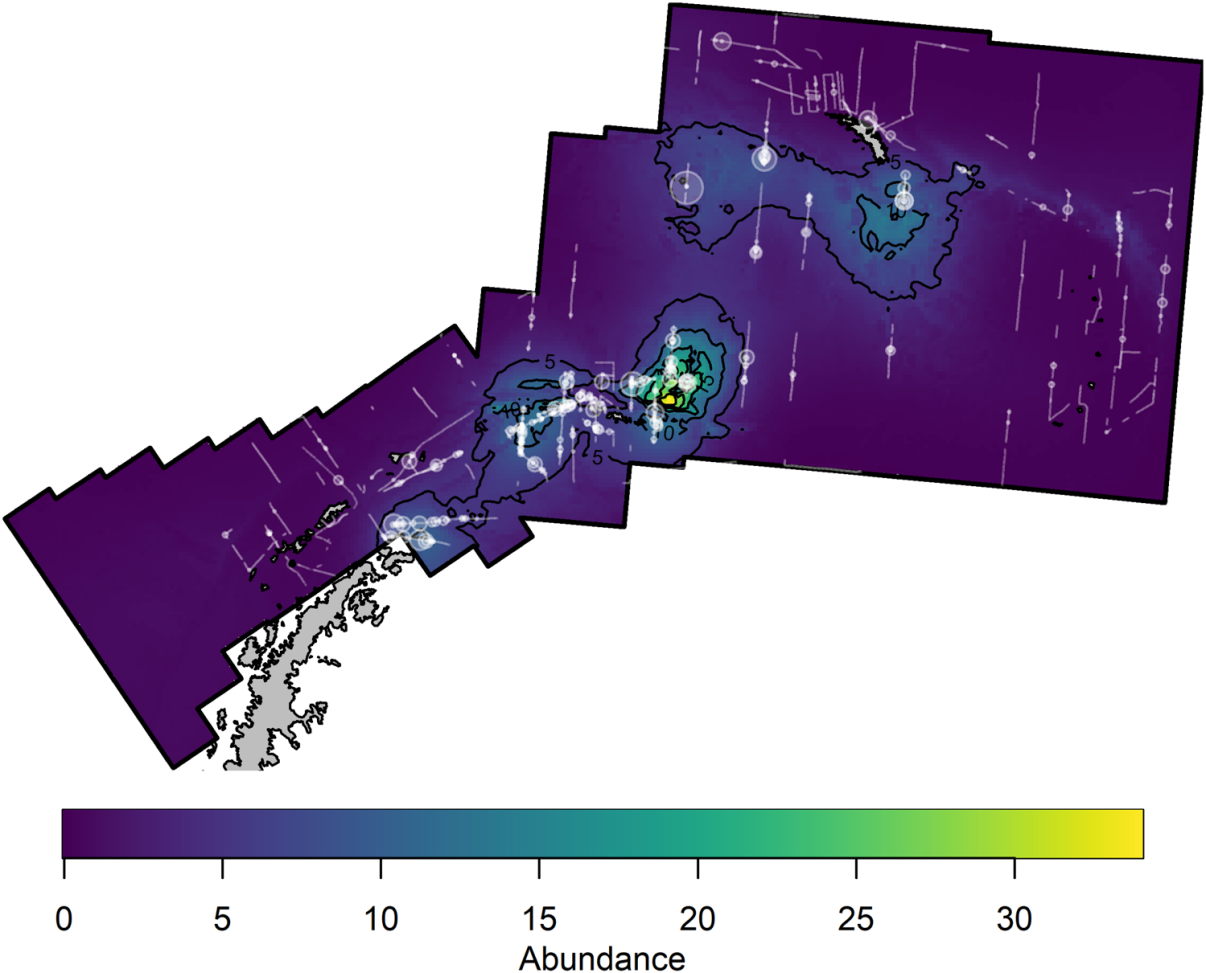
Predicted fin whale abundance across the full survey stratum covered during the 2019 survey from the density surface model. Numbers represent the number of whales per 100 km^2^.

The point estimate of abundance for this model was was 50,837, which is slightly lower than the corresponding design-based estimate, and also has a slightly lower coefficient of variation (0.136), and a 95% confidence of 38,966–66,324. The spatial variability in abundance predicted from this model is shown in Fig. 4, clearly highlighting the areas of high abundance around the South Orkney Islands, south of South Georgia, and around the tip of the Antarctic Peninsula.

**Table 6:** Results from density surface models, including abudance, CV and 95% confidence limits for the combined stratum.

Formula	Dev. expl	${\hat{N}}$	$cv({\hat{N}})$	$CI_{95}({\hat{N}})$
count~s(X, Y) + s(depth) + s(slope)	43.9%	50,837	0.136	38,966–66,324
count~s(X, Y) + s(depth)	43.7%	50,877	0.135	39,084–66,228
count~s(X, Y) + s(slope)	43.3%	50,353	0.135	38,716–65,487
count~s(X, Y)	42.8%	50,277	0.133	38,770–65,200

### Estimates of krill consumption

Estimates of daily and annual prey consumption are presented in Table 7, for individuals as well as the entire estimated fin whale population in the combined Scotia Sea and Antarctic Peninsula survey region combined. While estimates varied somewhat depending on the allometric scaling parameters used, there was nevertheless broad agreement, giving daily individual consumption estimates ranging from about 660 to 970 kg. Similarly, the energetically-derived daily individual consumption estimate (assuming 80% of annual energy requirements were met through feeding on krill for 180 days at the summer foraging grounds) was around 880 kg, and thus agreed well with the allometric estimates. At the population level, our simulations suggest annual krill consumtion rates in the wider Scotia Sea ranging from about 6.5 to 9.4 million tonnes, depending on the methods used.

**Table 7:** Simulated daily and annual consumption rates ($\hat{CR_d}$ and $\hat{CR_a}$) along with their 5% and 95% quantiles ($Q_{5-95}(\hat{CR_d})$ and $Q_{5-95}(\hat{CR_a})$), corresponding to the abundance estimate for the entire survey area based on the best supported model with covariates. All rates are given in metric tonnes. The columns $\alpha$ and $\beta$ refers to the allometric scaling parameters used (see methods section for further details).

Level	Method	$\alpha$	$\beta$	$\hat{CR_d}$	$Q_{5-95}(\hat{CR_d})$	$\hat{CR_a}$	$Q_{5-95}(\hat{CR_a})$
Individual	Allometric	0.177	0.783	0.972	0.714–1.219	175	128–219
	Allometric	1.660	0.559	0.774	0.623–0.912	139	112–164
	Allometric	0.420	0.670	0.665	0.511–0.808	120	92–145
	Allometric	0.123	0.800	0.815	0.594–1.026	147	107–185
	Energetic			0.882	0.765–0.993	159	138–179
					–		–
Population	Allometric	0.177	0.783	52,411	40,166–66,190	9,433,917	7,229,847–11,914,155
	Allometric	1.660	0.559	41,739	31,987–52,711	7,513,028	5,757,684–9,487,954
	Allometric	0.420	0.670	35,834	27,462–45,254	6,450,148	4,943,168–8,145,803
	Allometric	0.123	0.800	43,921	33,659–55,468	7,905,733	6,058,698–9,984,226
	Energetic			47,568	36,453–60,072	8,562,309	6,561,609–10,813,005

## Discussion

This study provides the most recent large-scale estimate of fin whale abundance in the Atlantic sector of the Southern Ocean, and suggests that this stock has undergone a dramatic increase over recent decades. The most recent prior survey of the same region, the SOWER-2000 survey yielded an estimated an abundance of roughly 4700 (cv: ~0.8) fin whales in the AP and SS strata combined ^9^. Even the lowest 95% confidence estimate for the same region from our study (38,770) exceeds the SOWER-2000 estimate by almost 10-fold. As acknowledged by^9^, the SOWER-2000 estimate should be regarded as an underestimate, due to poor weather conditions during the survey, especially in the Scotia Sea (SS) stratum. While we provide estimates from both design-based and model-based approaches, the latter were relatively simple, including no covariates for detection probability, and only a limited set of environmental covariates for density and abundance. These DSM models were mostly included as a way to check that our design-based estimates were not biased by assuming homogeneous fin whale distribution throughout the survey stratum. More in-depth spatial modeling should be carried out to examine potential links between fin whale density and a range of environmental covariates (e.g. sea surface temperature, sea surface height and chlorophyll-$\alpha$). In addition, since these data were collected as part of the 2019 Area 48 Survey for Antarctic krill^19^, we have the ability to examine how fin whale distribution correlates with spatial variations in krill biomass. While these more advanced analyses are underway, the estimates presented here nevertheless provides an important complement to existing survey estimates of fin whale abundance that deserve attention especially within the context of krill and broader ecosystem management in the Southern Ocean.

Our fin whale density estimates are in general agreement with recent studies^12,13^ covering smaller geographic ranges, which also suggest a significant increase in fin whale abundance in the Atlantic sector of the Southern Ocean in recent decades. However, due to a lack of large-scale surveys covering also the presumed fin whale summer distribution extending north of 60^∘^S, the current status and rate of population increase remains poorly quantified. Projecting forwards from the estimated population size of about 5500 whales in the 1990s within an area covering about 68% of the open ocean area south of 60^∘^S^36^, and assuming an intrinsic growth rate of 4%^37^, would result in a 2019 abundance of about 14,700 fin whales (using 1994 as the starting year, representing a 25-year interval). This estimate is lower than our lowest confidence limit of 38,770 from within a relatively smaller area. While estimates of plausible population growth rates do not exist for fin whales, Zerbini et al.^38^ estimated a maximum plausible rate of population increase of humpback whales of 11.8%. Assuming this rate for fin whales, the corresponding projections for 2019 based on the estimate in Branch et al.^36^ would be about 89,400 fin whales. Applying intrinsic population growth rates of 4 and 11.8% to the SOWER-2000 fin whale abundance estimates covering the Antarctic Peninsula and Scotia Sea strata used in our study, yields estimated 2019 population sizes of roughly 9,900 and 39,100 fin whales respectively. The higher of these two estimates is above our lowest 95% confidence limit of 38,770, and given the assumption that the SOWER-2000 estimate is too low suggests that our current estimates may not be unrealistic. This would suggest that fin whales in the Southern Ocean may have undergone a population increase similar to that reported for humpback whales in this region^17,18^. Our mean population estimate for the entire area (DSM model-based estimate:50,837) represents 24% of the modelled pre-whaling female-only fin whale abundance of 209,490 in the Atlantic sector^8^, but covers less than 1/3 of the longitudinal extent of this sector. It should be noted that fin whales are large-bodied sleek cetaceans capable of high sustained swimming speeds and therefore capable of covering large ranges. It is possible that some of the apparent increase observed in our survey area can be explained by a redistribution from other Southern Ocean sectors. To assess this, a complete circumpolar survey would be required, that also encompasses regions north of 60^∘^S.

While these calculations are associated with substantial uncertainty and rely on several untested underlying assumptions, they nevertheless provide an indication that Southern Ocean fin whales may be undergoing a remarkable increase towards estimated pre-whaling abundances, at least in the Scotia Sea region in the Southwest Atlantic. Such an increase may have profound implications for our understanding of the feeding ecology of cetaceans and the management of other living organisms in the Southern Ocean as well as the growing fishery for Antarctic krill. Our estimates of annual krill consumption by fin whales in the Scotia Sea of roughly 8,000,000 tonnes exceeds the 390,168 tonnes taken by the krill fishery in all of CCAMLR Area 48 in the 2019-2020 season ^39^ by about 20 times. While even the estimated annual krill consumption by fin whales using the SOWER-2000 abundance estimate (700,000 tonnes) is about 6 times higher than the commercial catches in Area 48 during the 1999/2000 season, it is several orders of magnitude lower than the estimated krill consumption in 2019, based on our abundance estimate. Furthermore, our 2019 estimate of krill consumption by fin whales within the survey area represents about 12.7% of the 2019 summer krill standing stock estimated from data collected during the same survey^19^. This is consistent with recent estimates by Baines et al.^40^ that baleen whales may consume 19-29% of the krill standing stock around the South Sandwich Islands.

These calculations are naturally based on several simplifications and assumptions, many of which remain untested, and our estimates of krill consumption should therefore be treated with some caution. It should also be noted that fin whales almost certainly do not feed exclusively on Antarctic krill in the Southern Ocean. For instance, previous studies have described spatial overlaps between fin whale aggregations and areas of high concentrations of the euphausiid *Thysanoessa macrura*^12^. While our assumption that 80% of annual energy requirements are covered by krill consumption at the feeding grounds in the Scotia Sea attempts to account for this to some degree, it is not based on any scientific evidence. Despite these knowledge gaps regarding feeding ecology and residence times of fin whales in the Southern Ocean, our estimates of fin whale abundance in the Scotia Sea agrees well with previous studies in smaller regions, suggesting a strong increase of this species across a substantial sector of the Southern Ocean that is also the center of the commercial krill fishery. This highlights the urgent need to improve our understanding of the ecological role of recovering cetacean populations in the Southern Ocean, particularly for fin whales. This, in turn, requires a re-evaluation of all available survey data for fin whales, and stresses the urgent need for further large-scale survey efforts that also cover regions north of 60^∘^S.

## Data Availability

The datasets used and/or analysed during the current study are available from the corresponding author on reasonable request.

## References

[1] Rocha RC, Clapham PJ, Ivashchenko YV (2014). Emptying the oceans: A summary of industrial Whaling catches in the 20th century. Mar. Fish. Rev..

[2] Allison, C. International Whaling Commission Catch Data Base v. 7.1. (2020).

[3] Matsuoka K (2003). Overview of minke whale sightings surveys conducted on IWC/IDCR and SOWER Antarctic cruises from 1978/79 to 2000/01. J. Cetacean Res. Manag.

[4] Miyashita, T., Kato, H. & Kasuya, T. Worldwide Map of Cetacean Distribution based on Japanese Sighting Data (Volume 1). *Natl. Res. Inst. Far Seas Fish. Shimizu, Shizuoka, Japan* **1**, 140 pp. (1995).

[5] Kasamatsu F, Joyce G, Ensor P, Mermoz J (1996). Current occurrence of baleen whales in Antarctic waters. Rep. Int. Whal. Comm..

[6] Cooke, J. G. *Balaenoptera physalus*. *IUCN Red List Threat. Species 2018* **e.T2478A50**, (2018). 10.2305/IUCN.UK.2018-2.RLTS.T2478A50349982.en

[7] Matsuoka, K. & Hakamada, T. Updated estimates of abundance south of 60oS for fin whales (Balaenoptera physalus) in the Antarctic, based on the SOWER CPIII data set, IWC/IDCR-SOWER special Volume. (In press). *J. Cetacean Res. Manag.* 9 pp.

[8] Tulloch VJD, Plagányi ÉE, Matear R, Brown CJ, Richardson AJ (2018). Ecosystem modelling to quantify the impact of historical whaling on Southern Hemisphere baleen whales. Fish Fish..

[9] Hedley, S. *et al.* Modelling whale distribution: a preliminary analysis of data collected on the CCAMLR-IWC Krill Synoptic Survey, 2000. *Sci. Comm. Int. Whal. Comm.* **SC53E9**, 1–38 (2001). http://nova.wh.whoi.edu/palit/Hedley%20et%20al_2001_Rep.%20Int.%20Whal.%20Comm._Modelling%20whale%20distribution%20a%20preliminary%20analysis%20of%20data%20collected%20on%20the%20CCAMLR-IWC%20Krill%20Synoptic%20Survey,%202000.pdf

[10] Joiris CR, Dochy O (2013). A major autumn feeding ground for fin whales, southern fulmars and grey-headed albatrosses around the South Shetland Islands, Antarctica. Polar Biol..

[11] Santora JA, Schroeder ID, Loeb VJ (2014). Spatial assessment of fin whale hotspots and their association with krill within an important Antarctic feeding and fishing ground. Mar. Biol..

[12] Herr H (2016). Horizontal niche partitioning of humpback and fin whales around the West Antarctic Peninsula: Evidence from a concurrent whale and krill survey. Polar Biol..

[13] Viquerat S, Herr H (2017). Mid-summer abundance estimates of fin whales *Balaenoptera physalus* around the South Orkney Islands and Elephant Island. Endanger. Species Res..

[14] Herr H (2022). Return of large fin whale feeding aggregations to historical whaling grounds in the Southern Ocean. Sci. Rep..

[15] Ryan C (2023). Commercial krill fishing within a foraging supergroup of fin whales in the Southern Ocean. Ecology.

[16] Johnston SJ, Zerbini AN, Butterworth DS (2011). A Bayesian approach to assess the status of Southern hemipshere humpback whales (*Megaptera novaeangliae*) with an application to Breeding Stock G. J. Cetacean Res. Manag..

[17] Zerbini AN (2019). Assessing the recovery of an Antarctic predator from historical exploitation. Roy. Soc. Open Sci..

[18] Baines M (2021). Population abundance of recovering humpback whales *Megaptera novaeangliae* and other baleen whales in the Scotia Arc. South Atlantic. Mar. Ecol. Prog. Ser..

[19] Krafft BA (2021). Standing stock of Antarctic krill (*Euphausia superba* Dana, 1850) (Euphausiacea) in the Southwest Atlantic sector of the Southern Ocean, 2018–19. J. Crustac. Biol..

[20] Watkins JL, Hewitt R, Naganobu M, Sushin V (2004). The CCAMLR 2000 Survey: A multinational, multi-ship biological oceanography survey of the Atlantic sector of the Southern Ocean. Deep Res. Part II Top Stud. Oceanogr..

[21] IWC Annex H (2016). Report of the Sub-Committee on Other Southern Hemisphere Whale Stocks. J. Cetacean Res. Manag..

[22] øien, N. *Norwegian Independent Linetransect Survey 1995. Interne notat, nr. 8 - 1995, Havforskningsinstituttet*. 58 pp (1995).

[23] Miller D L, Rexstad E, Thomas L, Laake J L, Marshall L (2019). Distance sampling in R. J. Stat. Softw.

[24] Laake, J., Borchers, D., Thomas, L., Miller, D. & Bishop, J. mrds: Mark-recapture distance sampling. (2020). https://cran.r-project.org/package=mrds

[25] R Core Team. R: A Language and Environment for Statistical Computing. (2020). https://www.r-project.org/

[26] Akaike H (1974). A new look at the statistical model identification. IEEE Trans. Automat. Contr..

[27] Miller DL, Burt ML, Rexstad EA, Thomas L (2013). Spatial models for distance sampling data: Recent developments and future directions. Methods Ecol. Evol..

[28] Miller, D. L. *et al.**dsm: Density Surface Modelling of Distance Sampling Data*. (2022). https://cran.r-project.org/package=dsm

[29] Hijmans, R. J. *raster: Geographic Data Analysis and Modeling*. (2023). https://cran.r-project.org/package=raster

[30] Smith L A, Link J S, Cadrin S X, Palka D L (2015). Consumption by marine mammals on the Northeast U.S. continental shelf. Ecol. Appl.

[31] Skern-Mauritzen M (2022). Marine mammal consumption and fisheries removals in the Nordic and Barents seas. ICES J. Mar. Sci.

[32] Hind AT, Gurney WS (1997). The metabolic cost of swimming in marine homeotherms. J. Exp. Biol..

[33] Braithwaite JE, Meeuwig JJ, Hipsey MR (2015). Optimal migration energetics of humpback whales and the implications of disturbance. Conserv. Physiol..

[34] Schaafsma FL (2018). Review: The Energetic Value of Zooplankton and Nekton Species of the Southern Ocean.

[35] Buckland ST, Rexstad EA, Marques TA, Oedekoven CS (2015). Distance Sampling: Methods and Applications.

[36] Branch T, Butterworth D (2001). Estimates of abundance south of 60^∘^S for cetacean species sighted frequently on the 1978/79 to 1997/98 IWC/IDCR-SOWER sighting surveys. J. Cetacean Res. Manag..

[37] Taylor, B. L., Chivers, S. J., Larese, J. & Perrin, W. F. Generation length and percent mature estimates for IUCN assessments of cetaceans. *Adm. Rep. LJ-07-01 Natl. Mar. Fish.* 24 pp (2007).

[38] Zerbini AN, Clapham PJ, Wade PR (2010). Assessing plausible rates of population growth in humpback whales from life-history data. Mar. Biol..

[39] CCAMLR. Fishery Report : Euphausia superba in Area 48. (2020).

[40] Baines M (2022). Ecological interactions between Antarctic krill (*Euphausia superba*) and baleen whales in the South Sandwich Islands region -Exploring predator-prey biomass ratios. Deep Res. Part I Oceanogr. Res. Pap..

